# Association of Candidate Genes with Response to Heat and Newcastle Disease Virus

**DOI:** 10.3390/genes9110560

**Published:** 2018-11-19

**Authors:** Kaylee Rowland, Perot Saelao, Ying Wang, Janet E. Fulton, Grant N. Liebe, Amy M. McCarron, Anna Wolc, Rodrigo A. Gallardo, Terra Kelly, Huaijun Zhou, Jack C. M. Dekkers, Susan J. Lamont

**Affiliations:** 1 Department of Animal Science, Iowa State University, Ames, IA 50011, USA; krowland@iastate.edu (K.R.); awolc@iastate.edu (A.W.); jdekkers@iastate.edu (J.C.M.D.); 2 Department of Animal Science, University of California, Davis, CA 95615, USA; psaelao@ucdavis.edu (P.S.); ucywang@ucdavis.edu (Y.W.); hzhou@ucdavis.edu (H.Z.); 3 Hy-Line International, Dallas Center, IA 50063, USA; jfulton@hyline.com (J.E.F.); GLiebe@hyline.com (G.N.L.); AMcCarron@hyline.com (A.M.M.); 4 School of Veterinary Medicine, University of California, Davis, CA 95616, USA; ragallardo@ucdavis.edu (R.A.G.); trkelly@ucdavis.edu (T.K.)

**Keywords:** haplotype, heat stress, Newcastle disease virus, commercial poultry, immune response

## Abstract

Newcastle disease is considered the number one disease constraint to poultry production in low and middle-income countries, however poultry that is raised in resource-poor areas often experience multiple environmental challenges. Heat stress has a negative impact on production, and immune response to pathogens can be negatively modulated by heat stress. Candidate genes and regions chosen for this study were based on previously reported associations with response to immune stimulants, pathogens, or heat, including: *TLR3*, *TLR7*, *MX*, MHC-B (major histocompatibility complex, gene complex), *IFI27L2*, *SLC5A1*, *HSPB1*, *HSPA2*, *HSPA8*, *IFRD1*, *IL18R1*, *IL1R1*, *AP2A2*, and *TOLLIP*. Chickens of a commercial egg-laying line were infected with a lentogenic strain of NDV (Newcastle disease virus); half the birds were maintained at thermoneutral temperature and the other half were exposed to high ambient temperature before the NDV challenge and throughout the remainder of the study. Phenotypic responses to heat, to NDV, or to heat + NDV were measured. Selected SNPs (single nucleotide polymorphisms) within 14 target genes or regions were genotyped; and genotype effects on phenotypic responses to NDV or heat + NDV were tested in each individual treatment group and the combined groups. Seventeen significant haplotype effects, among seven genes and seven phenotypes, were detected for response to NDV or heat or NDV + heat. These findings identify specific genetic variants that are associated with response to heat and/or NDV which may be useful in the genetic improvement of chickens to perform favorably when faced with pathogens and heat stress.

## 1. Introduction

Newcastle disease is considered the number one disease constraint to poultry production in low and middle-income countries [1]. In these areas, chickens often serve as important protein sources and commodities to be sold or traded. Pathogens are not the only challenge in small-scale poultry production in these resource-limited settings. Heat stress also has a negative impact [2]. Immune response is known to be negatively modulated by heat stress [3].

Historically, selective breeding for many traits has been very successful in poultry [4]. However, breeding for disease resistance and/or heat tolerance can be difficult due to the need for specific challenge facilities, the potential terminal nature of these traits, animal welfare concerns, biosafety concerns, and cost. The identification of genetic factors influencing disease resistance and/or heat tolerance would provide tools for improvement through selective breeding. The response to Newcastle disease virus (NDV) is known to be partly under genetic control [5,6,7,8,9]. Several scientists have reported differences in heat response between lines and breeds [10,11,12]. In this study, we identify genes and haplotype combinations within those genes that affect the response to NDV and/or heat challenge. Producing chickens that are genetically predisposed to perform better in the face of disease, vaccination, or environmental challenges (which often occur simultaneously in resource-limited settings) will ultimately lead to improved food and nutritional security and enhanced livelihoods in low-income countries with eventual global impacts.

Candidate genes and regions that were chosen for this study were based on previous reports of associations with disease response and/or a role in the heat stress response [13,14,15,16,17,18,19,20,21,22,23,24], including: *TLR3*, *TLR7*, *MX*, MHC-B (major histocompatibility complex, gene complex), *IFI27L2*, *SLC5A1*, *HSPB1*, *HSPA2*, *HSPA8*, *IFRD1*, *IL18R1*, *IL1R1*, *AP2A2*, and *TOLLIP*. Membrane receptors functioning in immune response (MHC, *IL18R1*, *IL1R1*, *IFRD1*) are responsible for recognizing foreign molecules. This recognition of antigens is crucial for immune function. *IFRD1* also has a role in muscle growth and differentiation [25]. Components of the innate immune system (*AP2A2*, *TLR3*, *TLR7*, *TOLLIP*, *MX*, *IFI27L2*) provide a first line of defense to a pathogen or virus. *TLR3*, *TLR7*, and *MX* have been directly implicated in antiviral response [13,21,26]. *TOLLIP* is part of the toll like receptor signaling pathway [27]. *AP2A2* is suggested to function in endocytosis, recognition, and processing of antigens [28]. *IFI27L2* functions in the apoptotic pathway [29].

Heat shock proteins and solute carriers (*HSPA2*, *HSPB1*, *HSPA8*, *SLC5A1*) play a role in maintaining homeostasis and buffering the negative impacts of hyperthermic conditions as well as disease challenges [30,31]. Heat shock proteins function in protein folding, intracellular trafficking, and managing proteins that are denatured by heat and other stresses, such as pathogens. Some heat shock proteins, such as *HSPA2*, are involved in binding antigens and presenting them to the immune system [32].

In this study, we used data from two treatment groups of a brown egg-laying commercial hybrid cross (HYB). One group (NDV) was challenged with a lentogenic strain of NDV; the other (heat + NDV) was also exposed to high ambient temperature before and during the lentogenic NDV challenge. Phenotypic responses to NDV or heat + NDV were measured; selected single nucleotide polymorphisms (SNPs) within 14 target genes or regions were genotyped and gene haplotypes were identified. The genotype effects of these gene haplotypes on phenotypic responses to either the NDV challenge or heat + NDV challenge were tested.

## 2. Materials and Methods

### 2.1. Animals and Husbandry

Pooled semen from 16 sires was used to inseminate 145 dams to produce three hatches of 360 mixed-sex chicks of a commercial brown egg layer line (Hy-line Brown, Hy-Line International) for this study. Birds were provided ad libitum access to feed and water throughout the study period and were reared following standard commercial husbandry practices. Initially, 23 h of light was provided, which was gradually decreased to 13.5 h of light by day 29. The birds were reared at thermo-neutral temperature (until day 14 in the heat + NDV group). In each of the three replicates (hatches), half (180) of the chicks were ground transported from Hy-Line International in Dallas Center, IA, USA to a BSL-2 facility in Ames, IA, USA for a pathogen challenge, and the other half (180) were shipped by air the same day to a BSL-2 facility in Davis, California, USA for a thermal and pathogen challenge. The families (sibs) were distributed as evenly as possible using known dam information across the two treatment groups (NDV and heat + NDV). Institutional Animal Care and Use Committees approved all of the animal procedures and care in this study (log #1-13-7490-G and #17853). Genomic DNA was isolated from whole, non-coagulated (EDTA) blood that was collected from the brachial vein of all chicks pre-challenge.

### 2.2. Experimental Design

**Figure 1:**
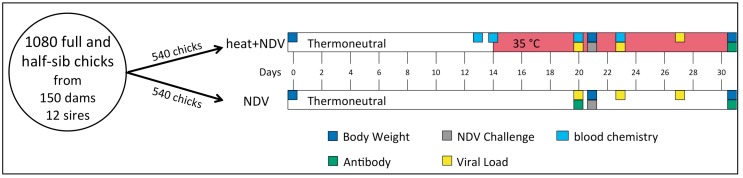
Experimental design. NDV: Newcastle disease virus.

The experiment was performed across three replicates (three hatches from the same dams and sires) of 180 birds for two treatment groups for 1080 birds in total. The chicks in the NDV treatment group were challenged with NDV. The chicks in the heat + NDV treatment group were subjected to the same NDV challenge with the addition of a heat challenge that was initiated before the NDV challenge and continued throughout the study. The experimental timeline for both of the treatment groups is depicted in Figure 1. High ambient temperature was included in the experimental protocol for the heat + NDV treatment group. Until day 14, the chicks were reared at thermo-neutral temperature and 60% humidity. At 14 days of age, the temperature was increased to 35 °C and was held until the conclusion of the trial (25 °C in the non-heated group). The optimum temperature for adult laying hens is 19–22 °C, with temperatures above this range resulting in heat stress [33]. For both treatment groups, at 21 days of age (0 dpi), the birds were inoculated with 10^8^ of 50% embryonic infectious dose (EID_50_) of live attenuated type B1 LaSota strain Newcastle disease vaccine in a volume of 200 μL. Virus propagation was detailed previously by Deist et al. [34]. The virus was administered via a natural, ocular-nasal route. Each eye and nares received ~50 μL of inoculum. The ND viral load was quantified at 20, 23, and 27 days of age and was thereafter designated as pre-challenge, 2 dpi, and 6 dpi, respectively. Anti-NDV antibody levels were measured on day 31 and were hereafter referred to as 10 dpi. Body weights were recorded on days 0, 21, and 31 of age. Previously, two distinct genome wide association studies (GWAS) (NDV and heat + NDV) associated 600 k genotypes from these birds with the NDV viral load, anti-NDV antibody, and growth rate phenotypes described herein [35,36].

### 2.3. Viral Load

To quantify the viral load, lachrymal fluid was collected from each bird two times, 2 dpi (*n* = 969) and 6 dpi (*n* = 965), as previously described by Deist et al. [34]. Viral RNA was isolated from each lachrymal fluid sample and was quantified via quantitative real-time PCR (qRT-PCR) in duplicate, as described by Deist et al. [34]. The mean viral RNA copy number was calculated per sample and was log10 transformed to achieve a distribution that was closer to normality.

### 2.4. Antibody

Sera samples were collected at 10 dpi (*n* = 916) for the quantification of anti-NDV antibody using an IDEXX NDV ELISA for chickens (IDEXX Laboratories, Inc., Westbrook, ME, USA). Each sample was quantified in duplicate and the average sample:positive (S/P) absorbance ratio was calculated per the manufacturer’s instructions and was log_10_ transformed to achieve a distribution that was closer to normality.

### 2.5. Growth Rate

Body weights were recorded in grams on days 0, 21 (0 dpi), and 31 (10 dpi). The pre NDV challenge growth rate (*n* = 991) was calculated as grams per day that were gained between days 0 and 21. The post NDV challenge growth rate (*n* = 969) was calculated as grams per day that were gained between days 21 and 31.

### 2.6. Heat-Related Phenotypes

Phenotypes related to the heat challenge were measured in the heat + NDV treatment group only. An i-STAT^®^ handheld blood analyzer (Abbott Laboratories, San Diego, CA, USA) was used to quantify 13 blood parameters: four chemistry/electrolyte parameters (concentrations of sodium (Na), potassium (K), ionized calcium (iCa), and glucose (Glu)); seven blood gas parameters (pH, carbon dioxide partial pressure (PCO_2_), oxygen partial pressure (PO_2_), total carbon dioxide (TCO_2_), bicarbonate (HCO_3_), base excess (BE), and oxygen saturation (sO_2_)); and two hematologic parameters (termed “hematocrit” (Hct) and “hemoglobin” (Hb) by i-STAT^®^). Blood gas and chemistry parameters were measured four times: day 13 (pre-heat), day 14 (4 h post-heat initiation), day 20 (6 days post-heat initiation), and day 23 (9 days post-heat initiation and 2 days post NDV infection).

### 2.7. Selection of Genes and Genotyping

Genes or genetic regions were selected for genotyping based on previously reported and unreported associations with heat stress and/or immune response [13,14,15,16,17,18,19,20,21,22,23,24]. The SNPs within genes were selected for known variation within the study population. The targeted genes were: *TLR3*, *TLR7*, *MX*, *IFI27L2*, *SLC5A1*, *HSPB1*, *HSPA2*, *HSPA8*, *IFRD1*, *IL18R1*, *IL1R1*, *AP2A2*, *TOLLIP*. The MHC-B region (gene complex) was genotyped using a 90 SNP panel that encompasses 38 genes within 210.00 bp, as described in [37]. Details on the SNPs that were genotyped, their genomic location (galGal5), and the potential impact of each SNP change are summarized in Table S1. The number of haplotypes found for each gene, length (bp) of the genome covered by the SNPs, and the number of SNPs that were tested to define each haplotype are given in Table 1.

Single nucleotide polymorphisms were identified by visual examination of available genome sequence data of DNA pools for the elite lines that were used to produce the commercial hybrid chicks that were used in the challenge [38], utilizing IGV software [39]. The most current chicken genome build (galGal4) and annotations (UCSC RefSeq 54) available at that time were used to align the genome sequences. Only SNPs that were identified within exons and that were predicted to impact amino acid sequences were chosen for genotyping. Subsequent genome builds and annotations resulted in changes in alignment and annotation such that some of the genotyped SNPs were no longer identified as located within an exon. SNPs were selected to encompass as much of each gene as possible. Details for SNPs within each gene based on galGal5 are given in Table S1.

Genotyping was performed as single SNP assays using fluorescence-based allele specific detection with KASP chemistry [40] and analysis with Kraken software (LGC, Hoddeston, UK). The SNP data of each gene for each bird was used to identify the haplotypes segregating in the population and the specific haplotypes that were held by each individual. Haplotypes were defined by co-segregation patterns of SNPs (Tables S2–S12). Any haplotype occurring less than 10 times (0.5%) was excluded from the analysis.

### 2.8. Data Analysis

Heritabilities for anti-NDV antibody level at 10 dpi, ND viral load at 2 and 6 dpi, and pre and post NDV challenge growth rate were estimated across both treatment groups. Heritabilities for these NDV challenge related phenotypes were previously estimated for the treatment groups separately and were reported in [35,36]. For heat challenge related phenotypes, heritability was estimated among the heat challenged animals only [41].

Heritabilities were estimated in ASReml 4 [42] using the following univariate animal model:

Y_ijk_ = µ + S_i_ + RR_j_ + A_k_ + e_ijk_, (1)

where Y is the dependent variable of the phenotype. Sex (S) and a combined variable of room and replicate (RR) were fitted as fixed effects. Room and replicate were treated as distinct between treatments so that the RR effect captured treatment effects as well. Random effects included animal genetic effects (A) with a genomic relationship matrix that were computed using genotypes of 338,814 quality-controlled SNPs from the 600 K SNP panel [35,36] for 946 animals following the procedure described by [43], and residuals (e). The random effect of dam was included for pre-challenge measurements of growth rate. Phenotypic variance was obtained by summing estimates of variance due to animal, residual, and dam (where applicable). Heritability was calculated as a ratio of the estimates of animal to phenotypic variance. Genetic correlations between traits that were measured in both the treatment groups were also estimated with a bivariate animal model in ASReml 4 fitting the same effects as described above.

Haplotype effects were tested for phenotypes with heritability estimates different from zero (estimate greater than two times the standard error). Two types of haplotype effects were tested, copy number (i.e., 0, 1, or 2 copies of any given haplotype) and combination (i.e., which two haplotypes are present for a given gene). Haplotype copy number effects were tested separately for each haplotype (*n* = 65) and haplotype combination effects (effect of each specific pair of haplotypes that exist for a given gene) were tested separately for each gene or region *(n* = 14) by including haplotype copy number as a covariate or haplotype combination as a fixed effect in the univariate animal model that was described for heritability estimation. For phenotypes that were measured in both of the treatment groups, the interaction of treatment with haplotype (copy number or combination) was added. Individual SNPs were also tested for effects on phenotypes. *p*-values were adjusted for multiple tests within the phenotype using the Benjamini-Hochberg correction [44].

## 3. Results and Discussion

### 3.1. Viral Load

**Figure 2:**
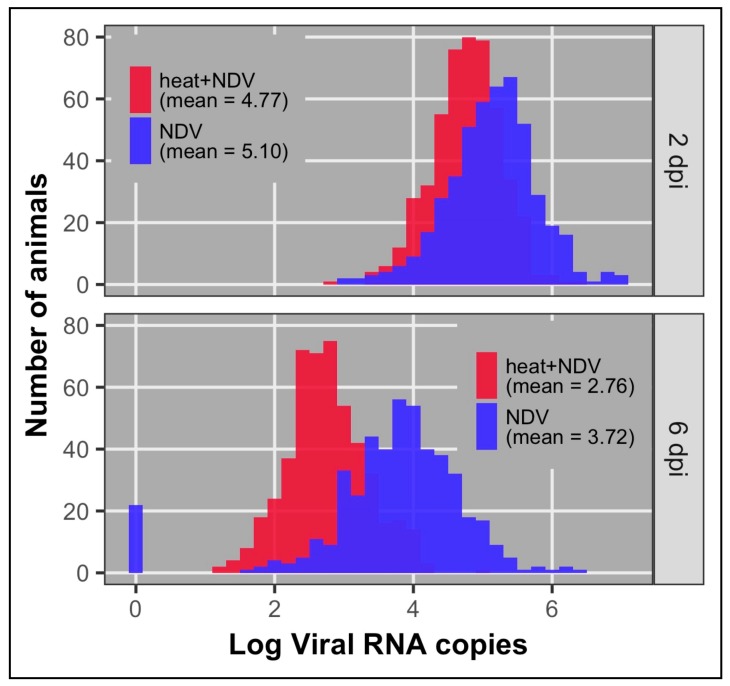
Viral load distributions at 2 and 6 dpi for both treatment groups.

None of the birds that were tested for the viral load pre-NDV challenge had detectable viral RNA, as expected (data not shown). Viral load was significantly higher via least squares means at 2 dpi compared to 6 dpi (*p* < 0.0001) in both of the treatment groups, indicating viral clearance between 2 and 6 dpi. Mean viral load at 2 and 6 dpi were significantly (*p* < 0.001) lower in the heat + NDV group compared to the NDV group (Table 2, Figure 2). The combined stressors of heat and NDV were expected to result in higher viral load in the heat + NDV treatment. We hypothesize that the higher environmental temperature in the heat + NDV treatment inactivated the shed virus to a greater degree than in the non-heated environment and, therefore, reduced the bird-to-bird virus transmission, resulting in lower viral loads in the heat + NDV group. The viral load means for the two groups are in Table 2.

Estimates of heritability for traits that were measured in both groups are reported in Table 2. The heritability estimate for viral load at 2 dpi for both groups (0.24 ± 0.06) was intermediate to separate estimates that were obtained from the NDV birds (0.32 ± 0.10) and the heat + NDV birds (0.17 ± 0.10) [35,36]. For 6 dpi, heritability for both groups was 0.09 (±0.04), while the estimates for the NDV and heat + NDV groups alone were not different from 0. Standard errors for the overall heritability estimates were also lower than the errors that were calculated separately by group, as expected with a larger data set. To our knowledge, these are the only reports of heritability for viral load of ND. Genetic correlation estimates of viral load between the two treatment groups had large standard errors (2 dpi, 0.51 ± 0.34; 6 dpi, 0.77 ± 0.56). No conclusion about genetic distinctiveness of the trait under two treatments can be made.

### 3.2. Antibody

**Figure 3:**
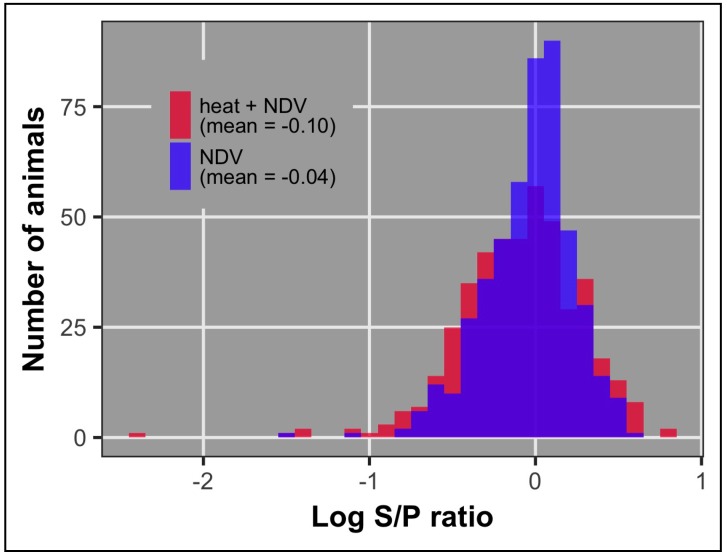
Antibody 10 dpi distribution by treatment group.

Mean anti-NDV antibody level at 10 dpi was significantly higher in the NDV group compared to the heat + NDV group (*p* = 0.013, Table 2, Figure 3). The mean anti-NDV antibody level for both groups is in Table 2. The heritability of the 10 dpi antibody level was intermediate for both groups (0.14 ± 0.05) compared to the NDV birds (0.24 ± 0.09) and the heat + NDV birds (0.04 ± 0.06), separately [35,36]. Heritability differences between the two treatment groups can be mostly explained by environmental differences. Environmental variation was much larger in the heat + NDV group compared to the NDV group. Genetic correlation estimates of anti-NDV antibody between the two treatment groups had a large standard error (0.26 ± 0.91). There is not enough power to estimate the genetic correlation with less than 500 birds in each treatment group when one of the groups has low heritability.

The heritability estimated for antibody levels that was reported by Lwelamira et al. in two Tanzanian chicken ecotypes that were measured two weeks post NDV vaccination (0.29) is slightly higher than our estimate for anti-NDV antibody at 10 dpi [45]. Peleg et al. also estimated a higher heritability (0.31) of antibody response to attenuated NDV at 12 dpi based on sire variance components in broilers [7].

### 3.3. Growth Rate

**Figure 4:**
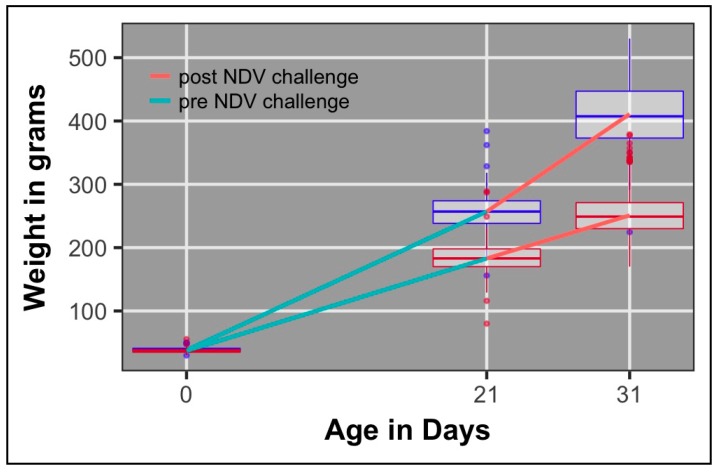
Body weights on days 0, 21, and 31 and regression lines for population average growth rate pre- and post-NDV challenge by treatment group. Red boxes indicate the heat + NDV treatment group, blue boxes indicate the NDV treatment group.

Growth rates, pre and post NDV challenge, were significantly higher in NDV birds compared to heat + NDV birds (*p* < 0.001, Table 2, Figure 4). Growth rate post challenge was significantly greater than growth rate pre challenge for both groups. The means of growth rates for both groups are in Table 2. Heritability for both growth rate periods was intermediate for both groups (pre: 0.40 ± 0.06 and post: 0.16 ± 0.05) compared to NDV birds (pre: 0.46 ± 0.11 and post: 0.21 ± 0.09) and heat + NDV birds (pre: 0.27 ± 0.09 and post: 0.11 ± 0.06) [35,36]. To our knowledge, these are the only reports of heritability for growth rate, under any condition, in layer-type birds. Genetic correlation of growth rate post-NDV between the two treatment groups had a large standard error (post-NDV, 0.22 ± 0.40). The genetic correlation of growth rate pre-NDV differed from 0 and 1, meaning that the growth trait that was measured in the two treatment groups shared some level of genetic control (0.66 ± 0.15), however that genetically it was not the same trait.

### 3.4. Heat-Related Phenotypes

Details of heritabilities and means for 13 i-STAT^®^ parameters and body temperature, measured in the heat + NDV treatment group to quantify the response to heat across four time-points, will be reported elsewhere [41]. Of these h^2^ estimates, eight were different from zero: HCO_3_, BE, and TCO_2_ on day 14 (acute heat); pH, PCO_2_, HCO_3_, BE, and TCO_2_ on day 23 (chronic heat). HCO_3_, BE, and TCO_2_ were the phenotypes with significant heritability across time. To our knowledge, the only other report of heritability estimates of i-STAT^®^ blood parameters under heat challenge was by Van Goor et al. [46]. Van Goor et al. did not report h^2^ estimates that were different from 0 for pH, PCO_2_, HCO_3_, BE, or TCO_2_ at 7 days post heat initiation.

### 3.5. Haplotype Effects

Of the tested genes, seven (*TLR7*, *MX*, *IFI27L2*, *SLC5A1*, *HSPA2*, *IFRD1*, *IL1R1*) had significant (*p* < 0.05) haplotype effects on at least one phenotype (Table 3). Significant effects were identified for both haplotype copy number effects (i.e., 0, 1, or 2 copies of any given haplotype) and haplotype combination effects (i.e., which two haplotypes are present for a given gene). For individual SNPs, only 12 of the many SNP effect tests (223 SNPs tested for each phenotype) were significant (Table S13). Haplotypes (large spans of information) can capture a higher degree of variation within a gene or region compared to SNPs (individual points of information). Therefore, it is not surprising that significant effects for haplotypes were found across multiple genes and phenotypes when individual SNP effects were scarce. For phenotypes that were measured in both groups, the interaction of treatment group and haplotype was significant in a few cases.

A consistent effect of *IFI27L2* was seen for antibody at 10 dpi (Table 3). Copy number effect for *IFI27L2*-H03 (H03 defined in Table S6) was significant in the NDV treatment group, as well as in the combined data of both treatment groups. Significant haplotype combination effects were also detected for *IFI27L2* in the NDV treatment group and in the combined data of both treatment groups. The shared significance and direction of effect between the analyses (NDV; heat + NDV), a lack of significance in the heat + NDV group, and a lack of significant haplotype by treatment interaction indicate the robustness of the action of *IFI27L2* during viral challenge. *Interferon alpha inducible protein 27* (*IFI27L2*) has been previously shown to be upregulated in response to several viruses, including NDV [13,47,48,49,50]. The current study is the first record of association of *IFI27L2* with antibody response and/or heat challenge. These results suggest that haplotype differences in the *IFI27L2* gene impact antibody production in response to NDV.

No effects were detected for viral load at 2 dpi in either group either separately or in the combined treatment groups. *SLC5A1* was associated with viral load at 6 dpi. Significant effects were identified for the *SLC5A1* haplotype pair combination in the NDV treatment group as well as in the combined data set (Table 3). *Solute carrier family 5 member 1* (*SLC5A1*) functions as a sodium/glucose cotransporter [51]. The implication of *SLC5A1* with viral load is novel. Expression of *SLC5A1* has been shown to be affected by hyperthermic conditions [52,53]. The shared significance and direction of the effect between the NDV challenge alone and the NDV challenge under heat, along with a lack of significance in the heat + NDV group and a lack of significant haplotype by treatment interaction, indicates the robustness of the role *SLC5A1* is playing in response to NDV.

Growth rate post-NDV challenge was associated with *IFRD1* (Table 3) and with *HSPA2* (Table 3). The effects of *IFRD1* haplotype combination and of *IFRD1*_H01 copy number were significant in the NDV group. *Interferon related developmental regulator 1* (*IFRD1*) functions in muscle growth and differentiation [25] and, importantly for laying hens, in the balance of bone formation and resorption [54]. Many quantitative trait loci that are associated with growth, body weight, and fat accumulation have been reported in the location of *IFRD1* [55]. *IFRD1* exhibits lower expression in the trachea and Harderian gland of Fayoumi (resistant) chickens compared to Leghorn (susceptible) chickens after NDV infection [26,34]. The Fayoumi and Leghorn chickens are inbred research lines representing resistant and susceptible models, respectively. Our finding of a significant effect of *IFRD1* haplotype on growth rate is in agreement with previous findings. The additional implication of *IFRD1* in bone formation and resorption is particularly relevant since the population studied was a commercial laying hen line. *HSPA2* haplotype combination effect on growth rate post-NDV challenge was significant in the combined data and in the heat + NDV group. *Heat shock 70 kDa protein* (*HSPA2*) functions as a chaperone and assists in protein folding during exposure to heat or other stresses [56]. *HSPA2* has also been implicated in antigen binding and presentation [57]. These two previously identified roles for *HSPA2* contribute to its effect, presented here, on growth rate post NDV challenge. Under heat challenge conditions, growth during the NDV challenge is impacted by *HSPA2*. Under thermoneutral conditions, growth during the NDV challenge is impacted by *IFRD1*. The interaction of the *IFRD1* haplotype combination with the treatment group was the only significant haplotype by treatment group interaction (adjusted *p* < 0.1, data not shown). This difference in gene effects between the two treatment groups highlights the potential impact of hypothermic challenge on response to disease.

Eight significant haplotype combination effects were found for blood component traits that were measured in the heat + NDV treatment group on day 23 (9 days post heat initiation and 2 days post NDV inoculation). *HSPA2* had a significant effect on Base Excess (BE), Bicarbonate (HCO_3_), and Total Carbon Dioxide (TCO_2_). *MX* had a significant effect on pH and BE. *IL1RL1* had a significant effect on pH and *TLR7* had a significant effect on BE. Considering the known functions and roles of these genes, it is likely that the effect of *HSPA2* is mainly due to the heat challenge and the effects of *MX*, *IL1RL1*, and *TLR7* are due to the viral challenge. *MX* and *TLR7* were previously reported to be upregulated in response to NDV infection in the trachea, spleen, lung, and Harderian gland of Leghorn and Fayoumi inbred research lines in a companion study [26,34,58,59]. To our knowledge, there are no other reports of hematologic, serum chemistry, and blood gas traits under viral challenge in chickens. No gene or haplotype effects were found for other blood component traits.

Previously, two distinct GWAS (NDV and heat + NDV) combined 600 k genotypes from these birds with the NDV viral load, anti-NDV antibody, and growth rate phenotypes described herein [35,36]. Both studies identified multiple SNPs that met a suggestive (20%) genomewide threshold. For antibody at 10 dpi, one QTL was identified in each treatment group (NDV: chr 21, heat + NDV: chr 1). For viral load at 2 dpi, one QTL on chromosome 1 was identified in the heat + NDV group (none in the NDV group). For viral load at 6 dpi, one QTL was identified in each treatment group, on chromosome 4 in the NDV group and on chromosome 24 in the heat + NDV group. No overlap in the location of significance (SNPs or haplotypes) was seen between the GWAS and the results of the targeted genotyping that are presented here. Quite possibly, more variation linked to causative variants was captured in the targeted candidate gene genotyping than was captured in the 600k data, likely due to the much closer distance between SNPs that was used for the candidate gene analysis. Many more tests were performed in the GWAS than the candidate gene analysis that is presented here. Therefore, multiple test corrections would have been more stringent for the GWAS, creating the possibility that any potential shared effects (between GWAS and targeted genotyping) were considered non-significant in the GWAS results due to correction. The results of the two GWAS, combined with the results of the targeted genotyping analysis reported here, will enhance our understanding, providing a holistic view of response to infection under thermoneutral and heat stressed conditions.

Although previous studies have reported that the expression of the 14 genes and regions that were evaluated here was impacted by NDV, heat, or other viral challenge [13,14,15,16,17,18,19,20,21,22,23,24], it is not altogether unsurprising that the current study did not find many links between the haplotypes (nucleotide variation) of the genes that were considered and the phenotypes that were quantified in this study. The multiple intermediary steps and factors that impact gene expression make it difficult to identify links between nucleotide variation and expression. In addition, little overlap exists in the genetic background of the lines that were used in all these studies (inbred vs. hybrid, research vs. commercial, etc.). The genetic background of the line used has a significant impact on the results of genetic studies. Therefore, it is essential to pair relevant genetic lines with the subject of the study.

It is important to add a note of caution in the interpretation of our results. Pre-challenge (day 13) body weight was significantly different between the treatment groups (facilities). At this early phase of the experiment, the groups did not differ in terms of applied treatments. However, the birds in the group that were intended to receive the heat + NDV treatment did have a longer transport distance to their facility and, thus, a longer delay in initial access to feed and water and potentially additional transport stressors. Therefore, phenotypic differences that were measured in the later phases (viral load and antibody), which were confounded with treatment facility, may have been influenced to some extent by facility-specific environmental differences other than the applied treatments. However, the RR fixed effect accounted for differences between the groups (facilities), intentional and unintentional, in the statistical model.

In summary, seventeen significant effects among seven genes (*TLR7*, *MX*, *IFI27L2*, *SLC5A1*, *HSPA2*, *IFRD1*, *IL1R1*) and seven phenotypes (growth rate post-NDV, viral load 6 dpi, antibody 10 dpi, BE, HCO_3_, TCO_2_, pH), were detected for gene haplotype copy number or haplotype combination on NDV and heat response. These gene effects provide increased knowledge of the genomic control of NDV and heat response and provide potential SNP targets for genomic selection. These findings, combined with those of companion studies, enhance the knowledge base that is needed for the genetic improvement of the performance of chickens that are faced with combined pathogen and environmental challenges.

## Figures and Tables

**Table 1:** Gene, haplotype, and single nucleotide polymorphism (SNP) summary.

Gene	Gene Name	Number of Haplotypes	Base Pairs ^1^	Number of SNPs
*TLR3*	*Toll like receptor 3*	3	4819	21
*TLR7*	*Toll like receptor 7*	3	1227	5
*MX*	*MX dynamin like GTPase 1*	5	20,492	34
MHC-B	Major histocompatibility complex B region ^2^	9	210,744	90
*IFI27L2*	*Interferon alpha inducible protein 27 like 1*	4	446	7
*SLC5A1*	*Solute carrier family 5 member 1*	5	9763	8
*HSPB1*	*Heat shock protein family B (small) member 1*	2	1739	5
*HSPA2*	*Heat shock 70 kDa protein 2*	4	1364	5
*HSPA8*	*Heat shock 70 kDa protein 8*	7	5747	6
*IFRD1*	*Interferon related developmental regulator 1*	3	3774	3
*IL18R1*	*Interleukin 18 receptor 1*	3	6563	3
*IL1R1*	*Interleukin 1 receptor type 1*	4	7979	7
*AP2A2*	*Adaptor related protein complex 2 subunit alpha 2*	9	45,285	15
*TOLLIP*	*Toll interacting protein*	4	26,625	14

^1^ Distance from first SNP to last SNP. ^2^ Gene complex encompassing 38 genes.

**Table 2:** Heritability (h^2^) and descriptive statistics of phenotypes measured in both of the treatment groups and separately in response to NDV (Newcastle disease virus).

Trait	Both Treatment Groups			NDV ^3^			Heat + NDV ^4^		
	h^2^ (SE)	N	Mean	h^2^ (SE)	N	Mean	h^2^ (SE)	N	Mean
Viral load 2 dpi ^1^	0.24 (0.06)	969	4.94	0.32 (0.10)	468	5.10	0.17 (0.10)	501	4.77
Viral load 6 dpi ^1^	0.09 (0.04)	965	3.22	0.18 (0.10)	470	3.72	0.11 (0.08)	495	2.76
Antibody 10 dpi ^1^	0.14 (0.05)	916	−0.07	0.24 (0.09)	448	−0.04	0.04 (0.06)	468	−0.10
Growth rate pre-challenge ^2^	0.40 (0.06)	991	8.62	0.46 (0.11)	473	10.4	0.27 (0.09)	518	6.93
Growth rate post-challenge ^2^	0.16 (0.05)	969	10.97	0.21 (0.09)	470	15.4	0.11 (0.06)	499	6.7

^1^ Phenotypes log10 transformed. ^2^ pre and post NDV challenge. ^3^ Referenced from [35]. ^4^ Referenced from [36]. SE: standard error.

**Table 3:** *p*-values for genes with significant haplotype combination effects and for additive effects of individual haplotypes.

Trait	Time Point	Analysis (Treatment Group)	Gene	*p*-Value	Adjusted *p*-Value	Haplotype with Significant Additive Effect	*p*-Value for Additive Effect	Adjusted *p*-Value for Additive Effect
Viral load	6 days post NDV	combined	*SLC5A1*	0.001	0.014			
		NDV	*SLC5A1*	0.01	0.14			
Antibody	10 days post NDV	combined	*IFI27L2*	0.002	0.028	H03 ^1^	0.003	0.18
		NDV	*IFI27L2*	0.01	0.14	H03 ^1^	0.001	0.06
Growth rate	post heat and NDV	combined	*HSPA2*	0.006	0.084			
		NDV	*IFRD1*	0.002	0.028	H01 ^2^	0.001	0.06
		heat + NDV	*HSPA2*	0.001	0.014			
BE	9 days post heat; 2 days post NDV	heat + NDV	*HSPA2*	0.001	0.014			
		heat + NDV	*TLR7*	0.021	0.098			
		heat + NDV	*MX*	0.017	0.098			
pH	9 days post heat; 2 days post NDV	heat + NDV	*MX*	0.004	0.056			
		heat + NDV	*IL1RL1*	0.011	0.077			
HCO_3_	9 days post heat; 2 days post NDV	heat + NDV	*HSPA2*	0.002	0.028			
TCO_2_	9 days post heat; 2 days post NDV	heat + NDV	*HSPA2*	0.003	0.042			

^1^*IFI27L2*-H03 defined in Table S6. ^2^*IFRD1*-H01 defined in Table S7.

## Supplementary Materials

The following are available online at http://www.mdpi.com/2073-4425/9/11/560/s1, Table S1: Genotyping details, SNP locations and impacts, Table S2: AP2A2 SNPs and haplotype configurations, Table S3: HSPA2 SNPs and haplotype configurations, Table S4: HSPA8 SNPs and haplotype configurations, Table S5: HSPB1 SNPs and haplotype configurations, Table S6: IFI27L2 SNPs and haplotype configurations, Table S7: IFRD1 SNPs and haplotype configurations, Table S8: IL1RL1 SNPs and haplotype configurations, Table S9: SLC5A1 SNPs and haplotype configurations, Table S10: TLR3 SNPs and haplotype configurations, Table S11: TLR7 SNPs and haplotype configurations, Table S12: TOLLIP SNPs and haplotype configurations, Table S13: P-values of individual SNPs with significant effect.
