# Supplementary material for: Association of Candidate Genes with Response to Heat and Newcastle Disease Virus
Source: Genes (Basel). 2018 Nov 19;9(11):560. doi: 10.3390/genes9110560 (PMC6267452; doi:10.3390/genes9110560)
Supplement: Supplementary file 1 [file genes-09-00560-s001.pdf]

Table S1: Genotyping details, SNP locations and impacts (based on galGal5)

Gene	SNP	Chr:bp	Location[1]	nt change	Codon Change	Amino Acid
AP2A2	00001	5:14,504,585	5' upstream	T>C	-	-
	i1-02199	5:14,502,020	Intron 1	G>C	-	-
	i1-05799	5:14,498,420	Intron 1	G>A	-	-
	i1-10304	5:14,493,915	Intron 1	C>G	-	-
	i2-14650	5:14,489,569	Intron 2	C>T	-	-
	i2-18015	5:14,486,204	Intron 2	T>A	-	-
	i3-21570	5:14,482,649	Intron 3	C>A	-	-
	i6-25342	5:14,478,877	Intron 6	C>T	-	-
	i9-28615	5:14,475,604	Intron 9	C>T	-	-
	1434	5:14,474,099	Exon 11	A>C	T**A**T>T**C**T	Y442S
	i13-31160	5:14,473,059	Intron 13	A>G	-	-
	i16-34011	5:14,470,208	Intron 16	G>A	-	-
	i20-37517	5:14,466,702	Intron 20	C>T	-	-
	i23-39719	5:14,464,500	Intron 23	C>T	-	-
	43919	5:14,460,300	3' downstream	T>C	-	-
HSP70	0258	5:52,785,824	Exon 1	G>A	TC**G**>TC**A**	S86S
	0909	5:52,785,173	Exon 1	C>A	GC**C**>GC**A**	A303A
	0985	5:52,785,097	Exon 1	C>T	**C**TT>**T**TT	L329F
	1044	5:52,785,038	Exon 1	G>A	AA**G**>AA**A**	K348K
	1622	5:52,784,460	Exon 1	C>A	G**C**C>G**A**C	A541D
HSPA8	I1-027	24:3,069,453	Intron 1	C>A	-	-
	0391	24:3,070,254	Exon 2	G>A	AA**G**>AA**A**	K25K
	1072	24:3,071,587	Exon 5	C>T	GA**C**>GA**T**	D252D
	1471	24:3,072,303	Exon 6	T>C	TC**T**>TC**C**	S385S
	1831	24:3,072,974	Exon 7	T>C	AA**T**>AA**C**	N505N
	3260	24:3,075,200	3' downstream	C>T	-	-
HSPB1	133	19:4,255,590	Exon 1	G>C	TG**G**>TG**C**	W42C
	280	19:4,255,443	Exon 1	C>T	GC**C**>GC**T**	A91A
	I1-1005	19:4,254,450	Intron 1	G>A	-	-
	459	19:4,253,765	Exon 3	T>A	G**T**G>G**A**G	V164E
	559	19:4,253,704	Exon 3	C>T	GT**C**>GT**T**	V184V
IFI27L2	161	23:4,295,436	Exon 2	G>T	**G**CC>**T**CC	A35S
	219	23:4,295,494	Exon 2	A>G	A**A**C>A**G**C	N54S
	291	23:4,295,816	Exon 3	T>G	G**T**T>G**G**T	V78G
	299	23:4,295,824	Exon 3	G>C	**G**TC>**C**TC	V81L
	312	23:4,295,837	Exon 3	C>T	T**C**C>T**T**C	S85F
	324	23:4,295,849	Exon 3	C>T	C**C**G>C**T**G	P89L
	326	23:4,295,851	Exon 3	G>C	**G**TC>**C**TC	V90L
	357	23:4,295,882	Exon 3	A>C	T**A**A>T**C**A	*100S
IFRD1	0319	1:26,863,605	Exon 2	G>A	A**G**T>A**A**T	S21N
	0390	1:26,863,534	Exon 2	C>G	**C**CC>**G**CC	P45A
	1038	1:26,859,831	Exon 8	G>C	**G**AT>**C**AT	D261H
IRIL1	202-0149	1:133,233,629	Exon 2	G>A	**G**TG>**A**TG	V18M
	202-0213	1:133,235,394	Exon 3	A>T	G**A**T>G**T**T	D39V
	202-0548	1:133,236,107	Exon 5	C>A	**C**TA>**A**TA	L151I
	202-0551	1:133,236,110	Exon 5	C>G	**C**AG>**G**AG	Q152E
	202-0572	1:133,236,131	Exon 5	T>C	**T**AT>**C**AT	Y159H
	202-0869	1:133,239,869	Exon 7	G>A	**G**AT>**A**AT	D258N
	202-1056	1:133,241,608	Exon 9	C>T	C**C**T>C**T**T	P320L
SLC5A1	0484	15:8,778,833	Exon 6	T>C	**T**TG>**C**TG	L162L

	0906	15:8,776,145	Exon 9	C>T	TG**C**>TG**T**	C302C
	I10-6	15:8,775,513	Intron 10	G>A	-	-
	1464	15:8,772,392	Exon 13	G>T	GA**G**>GA**T**	E488D
	1505	15:8,772,351	Exon 13	G>A	T**G**T>T**A**T	C502Y
	2995	15:8,769,777	Exon 15 (UTR)	C>T	-	-
	3190	15:8,769,582	Exon 15 (UTR)	A>G	-	-
	3702	15:8,769,070	Exon 15 (UTR)	T>C	-	-
TLR3	0050	4:60,925,710	Intron 1	T>C	-	-
	0095	4:60,925,755	Intron 1	G>A	-	-
	0203	4:60,925,863	Exon 2	A>T	GAT>GTT	D19V
	0274	4:60,925,934	Exon 2	A>C	AAA>CAA	K43Q
	0389	4:60,926,049	Exon 2	A>G	AAT>AGT	N81S
	0538	4:60,927,055	Exon 3	A>G	AGC>GGC	S131G
	0574	4:60,927,091	Exon 3	A/-	AAA>	frameshift
	0719	4:60,928,079	Exon 4	A>C	AAC>CAC	N174H
	0892	4:60,928,252	Intron 5	T>G	-	-
	1000	4:60,928,360	Exon 6	A>T	**A**CT>**T**CT	T201S
	1197	4:60,928,557	Exon 6	G>C	AG**G**>AG**C**	R266S
	1247	4:60,928,607	Exon 6	A>G	G**A**G>G**G**G	E283G
	1538	4:60,928,898	Exon 6	G>A	A**G**G>A**A**G	R380K
	1781	4:60,929,141	Exon 6	C>T	G**C**G>G**T**G	A461V
	1795	4:60,929,155	Exon 6	G>C	**G**AC>**C**AC	D466H
	1936	4:60,929,296	Exon 6	G>T	**G**CT>**T**CT	A513S
	2108	4:60,929,468	Exon 6	C>T	G**C**T>G**T**T	A570V
	2299	4:60,929,659	Exon 6	A>T	**A**CT>**T**CT	T634S
	2356	4:60,929,716	Exon 6	G>A	**G**AA>**A**AA	E653K
	2397	4:60,929,757	Exon 6	A>G	AT**A**>AT**G**	I666M
	2545	4:60,930,529	Exon 7	G>A	**G**AT>**A**AT	D716N
TLR7	0417	1:122,859,432	Exon 2	C>T	**C**GT>**T**GT	R030C
	0666	1:122,859,183	Exon 2	G>A	**G**TC>**A**TC	V113I
	0708	1:122,859,141	Exon 2	A>T	**A**CA>**T**CA	T127S
	0781	1:122,859,068	Exon 2	G>A	C**G**T>C**A**T	R151H
	3169	1:122,856,680	Exon 2	A>C	C**A**G>C**C**G	Q947P
TOLLIP	00001	5:14,041,383	5' upstream	G>A	-	-
	i-02190	5:14,044,248	Intron 1	A>G	-	-
	i-04007	5:14,046,065	Intron 1	T>C	-	-
	i-07212	5:14,049,270	Intron 1	C>T	-	-
	i2-09101	5:14,051,159	Intron 2	A>G	-	-
	0365	5:14,053,920	Exon 3	T>G	**T**AT>**G**AT	Y83D
	i3-12440	5:14,054,498	Intron 3	A>G	-	-
	i4-14397	5:14,056,455	Intron 4	A>G	-	-
	i5-17066	5:14,059,124	Intron 5	A>G	-	-
	i5-19496	5:14,061,554	Intron 5	A>C	-	-
	i5-22503	5:14,064,561	Intron 5	A>G	-	-
	0767	5:14,065,571	Exon 6	A>G	**A**CA>**G**CA	T217A
	0894	5:14,065,698	Exon 6	A>G	A**A**C>A**G**C	N259S
	25950	5:14,068,008	3' downstream	G>A	-	-

[1]Location relative to gene elements

Table S2: AP2A2 SNPs and haplotype configurations

	SNPs															
	00001	i1-02199	i1-05799	i1-10304	i2-14650	i2-18015	i3-21570	i6-25342	i9-28615	1434	i13-31160	i16-34011	i20-37517	i23-39719	43919	Concatenated Haplotypes
H01	T	C	G	C	T	T	C	T	C	A	G	G	T	T	T	TCGCTTCTCAGGTTT
H02	T	C	G	C	T	A	C	T	T	A	A	A	C	T	T	TCGCTACTTAAACTT
H03	C	G	A	G	C	T	A	C	C	A	G	A	T	T	C	CGAGCTACCAGATTC
H04	C	G	A	G	C	T	C	C	C	A	A	G	C	T	T	CGAGCTCCCAAGCTT
H05	T	C	G	G	C	T	C	C	C	A	A	G	C	C	T	TCGGCTCCCAAGCCT
H06	C	G	A	G	C	T	C	T	C	A	G	G	T	T	T	CGAGCTCTCAGGTTT
H07	C	G	G	C	T	A	A	T	T	A	A	G	C	C	T	CGGCTAATTAAGCCT
H08	C	G	A	G	C	T	A	C	C	A	A	G	C	C	T	CGAGCTACCAAGCCT
H09	C	G	A	G	C	T	A	T	T	A	G	A	C	T	C	CGAGCTATTAGACTC
H10	T	C	G	C	T	A	C	T	T	A	A	A	T	T	T	TCGCTACTTAAATTT
H11	C	G	G	C	T	A	A	T	T	A	G	A	C	T	T	CGGCTAATTAGACTT
H12	C	G	A	G	C	T	C	T	T	A	A	A	C	T	T	CGAGCTCTTAAACTT
H13	C	C	G	G	C	T	C	C	C	A	A	G	C	C	T	CCGGCTCCCAAGCCT
H14	C	G	A	G	T	A	C	C	C	A	A	G	C	T	T	CGAGTACCCAAGCTT
H15	C	C	G	G	C	A	C	C	C	A	A	A	C	T	T	CCGGCACCCAAACTT
H16	C	G	A	G	C	A	C	C	C	A	A	G	C	T	T	CGAGCACCCAAGCTT
H17	T	C	G	C	T	T	C	T	C	A	G	A	C	T	T	TCGCTTCTCAGACTT
H18	C	G	A	G	C	T	A	T	T	A	G	A	T	T	C	CGAGCTATTAGATTC

Table S3: HSP70 SNPs and haplotype configurations

	SNPs					
	0258	0909	0985	1044	1622	Concatenated Haplotypes
H01	G	C	C	A	C	GCCAC
H02	A	C	C	G	C	ACCGC
H03	G	C	C	G	C	GCCGC
H04	A	C	C	A	C	ACCAC
H05	G	A	C	G	C	GACGC

Table S4: HSPA8 SNPs and haplotype configurations

	SNPs						
	I1-027	0391	1072	1471	1831	3260	Concatenated Haplotypes
H01	A	G	T	T	T	T	AGTTTT
H02	C	G	C	T	C	T	CGCTCT
H03	A	G	C	C	T	C	AGCCTC
H04	A	G	C	C	T	T	AGCCTT
H05	C	G	C	T	T	T	CGCTTT
H06	C	G	C	T	T	C	CGCTTC
H07	C	G	T	C	C	C	CGTCCC
H08	A	G	T	T	T	C	AGTTTC
H09	A	G	C	T	T	C	AGCTTC
H10	C	A	T	T	T	C	CATTTC
H11	C	G	C	T	C	C	CGCTCC

Table S5: HSPB1 SNPs and haplotype configurations

	SNPs					
	133	280	I1-1005	459	559	Concatenated Haplotypes
H01	G	C	G	T	C	GCGTC
H02	G	C	G	T	T	GCGTT
H03	G	T	A	T	T	GTATT
H04	G	C	A	T	T	GCATT

Table S6: IFI27L2 SNPs and haplotype configurations

	SNPs							
	161	219	291	299	324	326	357	Concatenated Haplotypes
H01	G	A	T	G	C	G	A	GATGCGA
H02	G	A	T	G	T	C	A	GATGTCA
H03	G	G	G	G	T	C	A	GGGGTCA
H04	G	A	G	G	T	C	A	GAGGTCA
H05	T	A	G	C	T	C	C	TAGCTCC
H06	G	A	G	G	C	G	C	GAGGCGC
H07	G	A	G	G	C	G	A	GAGGCGA

Table S7: IFRD1 SNPs and haplotype configurations

	SNPs			
	0319	0390	1038	Concatenated Haplotypes
H01	G	C	G	GCG
H02	G	G	G	GGG
H03	A	G	G	AGG

Table S8: IL1RL1 SNPs and haplotype configurations

	SNPs							
	202-0149	202-0213	202-0548	202-0551	202-0572	202-0869	202-1056	Concatenated Haplotypes
H01	G	T	A	C	C	G	C	GTACCGC
H02	G	T	C	C	T	A	T	GTCCTAT
H03	G	T	C	C	T	A	C	GTCCTAC
H04	A	A	C	C	T	A	T	AACCTAT
H05	G	A	C	G	T	A	T	GACGTAT
H06	G	T	A	C	C	A	T	GTACCAT
H07	G	A	A	C	C	A	T	GAACCAT
H08	G	A	C	C	T	A	T	GACCTAT

Table S9: SLC5A1 SNPs and haplotype configurations

	SNPs								
	0484	0906	I10-6	1464	1505	2995	3190	3702	Concatenated Haplotypes
H01	T	C	G	G	G	C	G	T	TCGGGCGT
H02	C	C	G	G	G	C	G	T	CCGGGCGT
H03	T	T	G	G	G	C	G	T	TTGGGCGT
H04	T	C	A	G	G	C	A	C	TCAGGCAC
H05	C	C	G	G	G	C	A	T	CCGGGCAT
H06	T	C	G	G	G	C	A	T	TCGGGCAT
H07	T	C	G	G	G	T	G	T	TCGGGTGT
H08	T	T	G	G	G	C	A	T	TTGGGCAT
H09	C	C	G	G	G	C	A	C	CCGGGCAC
H10	T	C	G	G	G	C	A	C	TCGGGCAC

Table S10: TLR3 SNPs and haplotype configurations

	SNPs																					
	0050	0095	0203	0274	0389	0538	0574	0719	0892	1000	1197	1247	1538	1781	1795	1936	2108	2299	2356	2397	2545	Concatenated Haplotypes
H01	T	G	A	A	A	A	A	A	T	A	C	G	A	C	G	G	T	T	G	A	G	TGAAAAAATACGACGGTTGAG
H02	C	G	A	A	G	A	A	C	T	A	C	G	A	C	C	G	T	T	G	A	G	CGAAGAACTACGACCGTTGAG
H03	C	G	A	A	A	A	A	A	T	A	G	G	A	T	G	G	C	A	G	A	G	CGAAAAAATAGGATGGCAGAG
H04	C	G	T	C	A	A	A	C	T	A	C	G	A	C	C	T	T	T	G	A	G	CGTCAAACTACGACCTTTGAG
H05	C	G	T	C	A	A	A	A	T	A	C	G	A	C	G	G	C	A	G	A	G	CGTCAAAATACGACGGCAGAG
H06	C	G	T	A	A	A	A	A	T	A	G	G	A	T	G	G	C	A	G	A	G	CGTAAAAATAGGATGGCAGAG
H07	C	G	A	A	A	G	A	A	T	A	G	G	A	T	G	G	C	A	G	A	G	CGAAAGAATAGGATGGCAGAG
H08	T	G	A	A	A	A	A	A	T	A	G	G	A	T	G	G	C	A	G	A	G	TGAAAAAATAGGATGGCAGAG
H09	C	G	A	A	A	A	A	A	T	A	G	G	G	C	G	G	T	T	G	A	G	CGAAAAAATAGGGCGGTTGAG
H10	C	G	A	A	A	A	A	A	T	A	C	G	A	C	G	G	T	T	G	A	G	CGAAAAAATACGACGGTTGAG

Table S11: TLR7 SNPs and haplotype configurations

	SNPs					
	0417	0666	0708	0781	3169	Concatenated Haplotypes
H01	C	A	T	G	A	CATGA
H02	C	G	A	G	A	CGAGA
H03	C	G	A	A	A	CGAAA
H04	C	A	A	G	A	CAAGA

Table S12: TOLLIP SNPs and haplotype configurations

	SNPs														
	00001	i-02190	i-04007	i-07212	i2-09101	0365	i3-12440	i4-14397	i5-17066	i5-19496	i5-22503	0767	0894	25950	Concatenated Haplotypes
H01	A	A	C	C	A	T	A	G	G	C	G	G	A	A	AACCATAGGCGGAA
H02	G	G	C	C	G	T	G	G	G	A	A	G	A	A	GGCCGTGGGAAGAA
H03	G	A	C	C	G	T	G	G	G	A	A	G	A	A	GACCGTGGGAAGAA
H04	A	A	C	T	A	T	G	G	G	C	G	G	A	A	AACTATGGGCGGAA
H05	G	G	T	C	A	T	A	A	G	A	A	G	A	A	GGTCATAAGAAGAA
H06	G	G	T	C	A	T	G	G	A	A	G	G	G	G	GGTCATGGAAGGGG
H07	G	A	T	C	G	T	G	G	G	A	A	G	A	A	GATCGTGGGAAGAA
H08	A	A	T	C	A	T	A	A	A	A	A	G	A	A	AATCATAAAAAGAA
H09	G	G	T	T	A	T	G	G	G	C	G	G	A	A	GGTTATGGGCGGAA
H10	A	A	C	C	A	T	A	A	A	A	G	G	A	G	AACCATAAAAGGAG
H11	A	A	T	C	A	T	A	A	A	C	G	G	A	A	AATCATAAACGGAA
H12	A	G	C	T	A	T	A	A	A	A	G	G	A	A	AGCTATAAAAGGAA
H13	A	A	C	C	G	T	G	G	G	A	A	G	A	A	AACCGTGGGAAGAA
H14	A	A	T	C	G	T	G	G	G	A	A	G	A	A	AATCGTGGGAAGAA
H15	G	G	T	C	A	T	A	G	G	C	G	G	A	A	GGTCATAGGCGGAA
H16	A	G	T	C	A	T	A	A	G	A	A	G	A	A	AGTCATAAGAAGAA
H17	G	A	T	C	A	T	G	G	A	C	A	G	A	G	GATCATGGACAGAG

Table S13: P-values of individual SNPs with significant effect

Time-point	Treatment	Trait	Gene	SNP	p-value	adjusted p-value
D23	9 days post heat; 2 days post NDV	pH	MX	PRO	0.002	0.0269
			MX	PRO2	0.002	0.0269
			MX	122	0.002	0.0269
			MX	125	0.002	0.0269
			MX	156	0.002	0.0269
			MX	351	0.001	0.0269
			MX	605	0.002	0.0269
			MX	1455	0.002	0.0269
			MX	1545	0.002	0.0269
			MX	1248	0.003	0.0363
			MHC	56	0.005	0.0465
			IFRD1	0319	0.005	0.0465
